# Supplementary material for: Curcumin effect on Acanthamoeba triangularis encystation under nutrient starvation
Source: PeerJ. 2022 Jul 4;10:e13657. doi: 10.7717/peerj.13657 (PMC9261923; doi:10.7717/peerj.13657)
Supplement: Supplemental Information 1 — Figure S1.Curcumin IC50 against A. triangularis trophozoites. The trophozoites were culture in PYG medium and treated with different concentrations of Curcumin. After 24 h treatment, parasite viability was analyzed by PrestoBlue® reagent and the fluorescence signal was measured by microplate reader. The IC50 was then calculated by Prism 5 software. The experiment was done with three independent experiments and data was represented as mean ± SD in µg/mL. Figure S2.Proportion of A. triangularis forms under starved conditions. Representative data of A. triangularis treated with 50 µg/mL Curcumin or 1 mM 3MA under starvation for 24 h. The parasite forms were quantified under light microscopy and represented as a percentage. Figure S3. Analysis of vacuole formation on surviving trophozoites. (A) A. triangularis trophozoites were cultured in starvation medium and treated with 50 µg/mL curcumin or 1 mM 3MA. (B) A. triangularis trophozoites were cultured in full medium and treated with 50 µg/mL curcumin. At least 200 cells per condition were examined for trophozoites containing vacuoles regardless of their number and size and represented as mean percentage ± SD. Data were obtained from 3 independent experiments. Figure S4.Gel electrophoresis of PCR products. Conventional PCR was performed to validate a primer specificity against A. triangularis DNA. The PCR product was run on 1.5% agarose gel. The primers targeting autophagy genes i.e. ATG16, ATG12, ATG8b, ATG3; encystation-related genes i.e. cellulose synthase (CS), serine proteinase (SP); apoptosis-related genes i.e. metacaspase (MCA), interleukin-1 converting enzyme-like protease (IL) including 18S rRNA were labelled on top of the gel. The first lane was a DNA ladder in bp (DM2300, SMOBIO®, Hsinchu, Taiwan) and the second lane was a negative control (Neg). Figure S5.Transcriptional expression of A. triangularis autophagy-related genes after 3MA treatment under starved condition. The trophozoites were cultured in PAS supp [file peerj-10-13657-s001.docx]

**Supplementary materials for:**

Curcumin effect on *Acanthamoeba triangularis* encystation under nutrient starvation

Rachasak Boonhok^1^_*_, Suthinee Sangkanu^2^, Suganya Phumjan^1^, Ramita Jongboonjua^1^, Nawarat Sangnopparat^1^, Pattamaporn Kwankaew^1^, Aman Tedasen^1^, Lim Chooi Ling^3^, Maria de Lourdes Pereira^4^, Mohammed Rahmatullah^5^, Polrat Wilairatana^6^*, Christophe Wiart^7^, Karma G. Dolma^8^, Alok K. Paul^9^, Madhu Gupta^10^, Veeranoot Nissapatorn^2^*

**
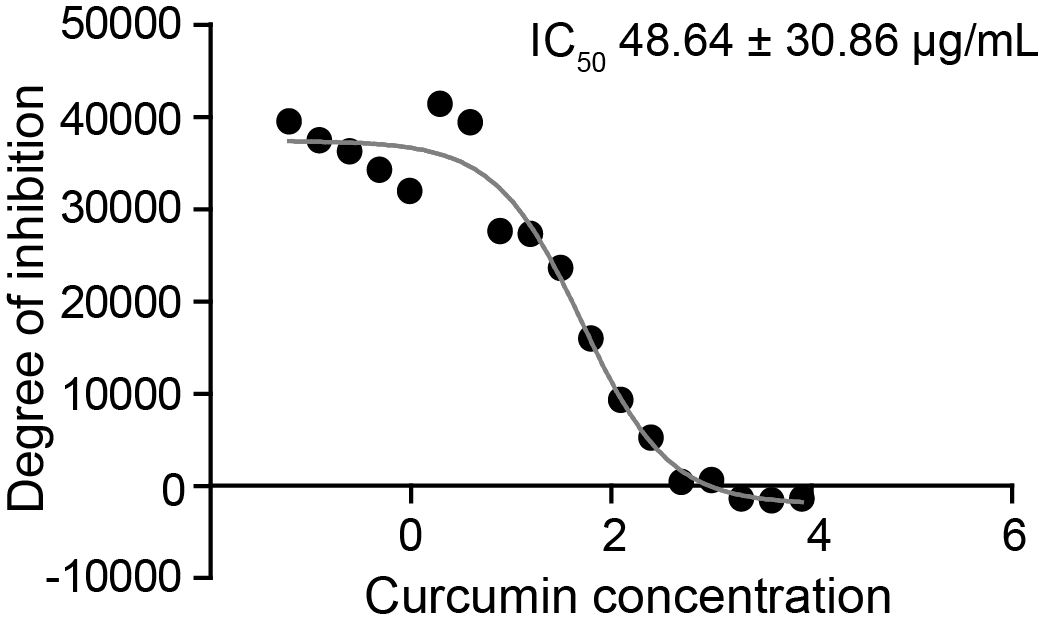
**

**Figure S1.** **Curcumin IC_50_ against *A. triangularis* trophozoites.** The trophozoites were culture in PYG medium and treated with different concentrations of Curcumin. After 24 h treatment, parasite viability was analyzed by PrestoBlue^®^ reagent and the fluorescence signal was measured by microplate reader. The IC_50_ was then calculated by Prism 5 software. The experiment was done with 3 independent experiments and data was represented as mean ± SD in µg/mL.


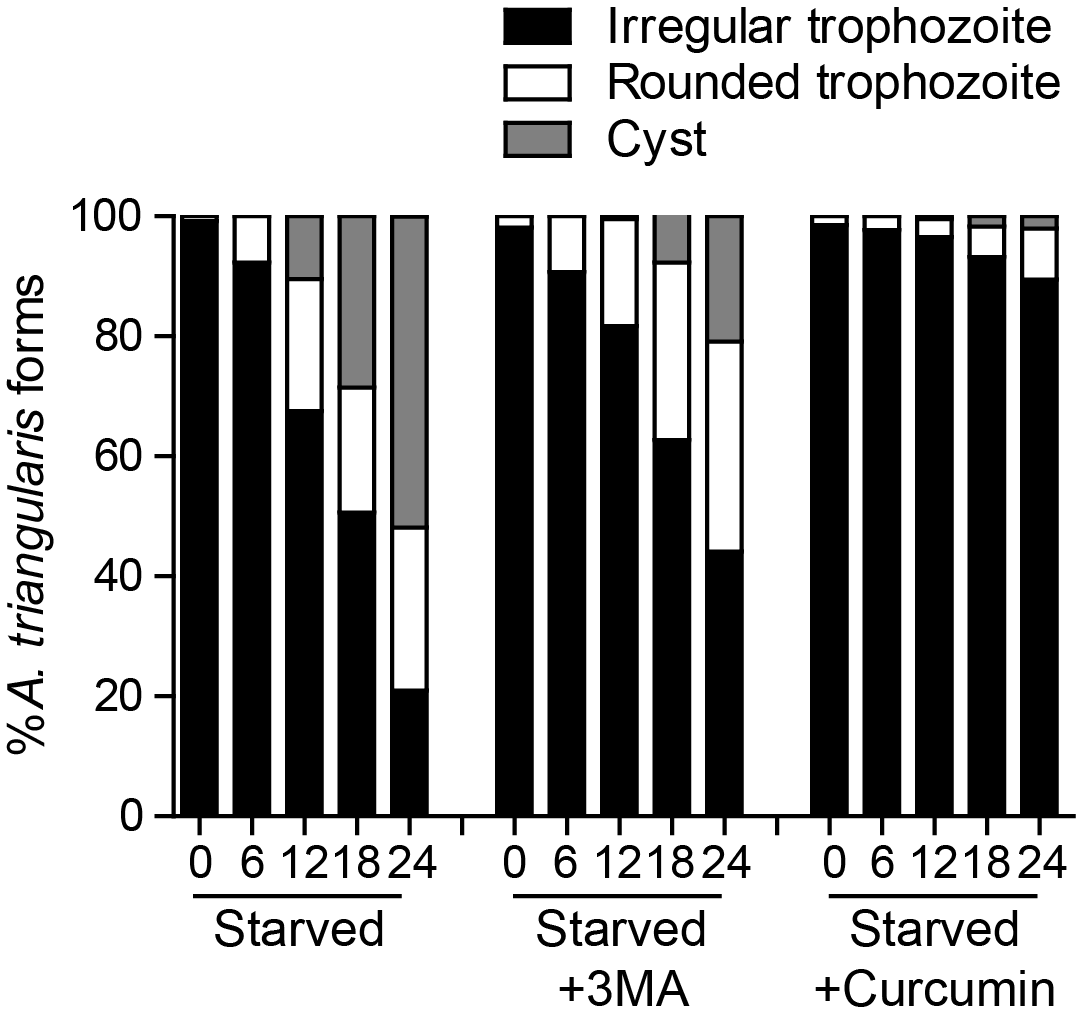


**Figure S2.** **Proportion of *A. triangularis* forms under starved conditions.** Representative data of *A. triangularis* treated with 50 µg/mL Curcumin or 1 mM 3MA under starvation for 24 h. The parasite forms were quantified under light microscopy and represented as a percentage.


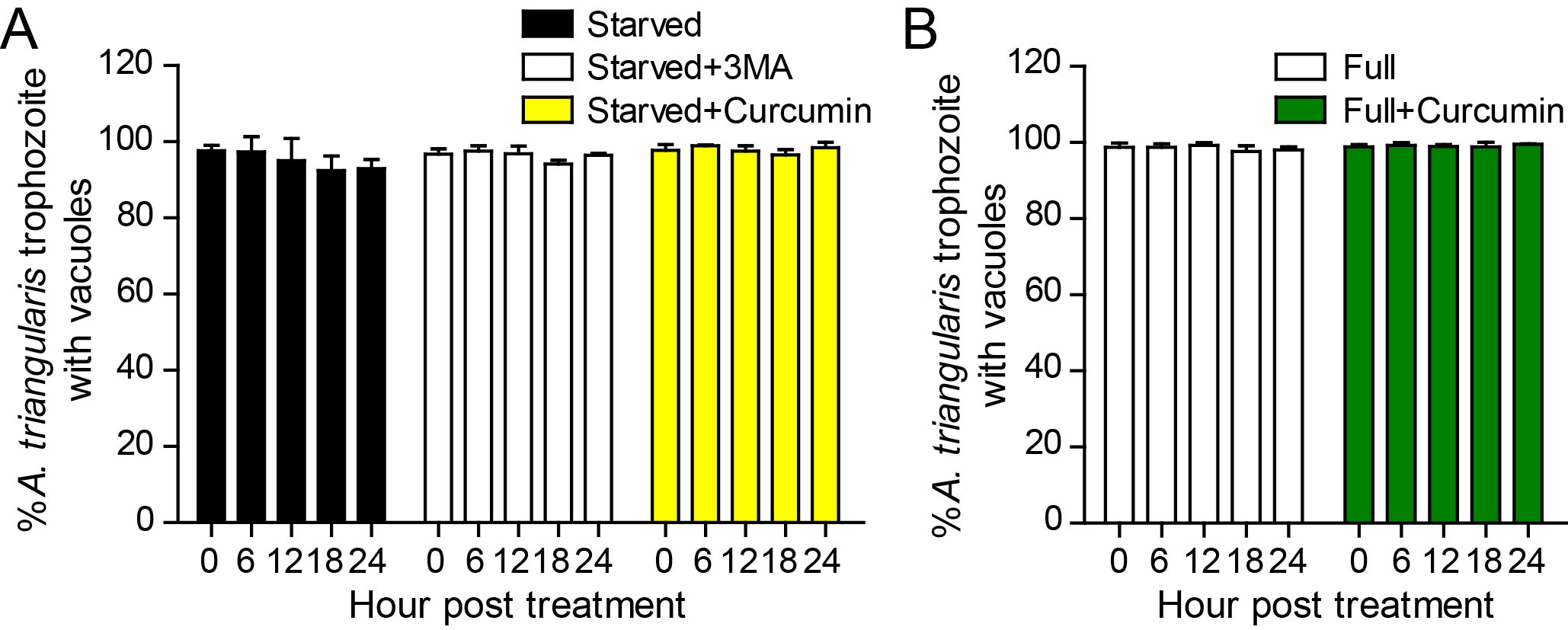


**Figure S3**. **Analysis of vacuole formation on surviving trophozoites.** **(A)** *A. triangularis* trophozoites were cultured in starvation medium and treated with 50 µg/mL curcumin or 1 mM 3MA. **(B)** *A. triangularis* trophozoites were cultured in full medium and treated with 50 µg/mL curcumin. At least 200 cells per condition were examined for trophozoites containing vacuoles regardless of their number and size and represented as mean percentage ± SD. Data were obtained from 3 independent experiments.


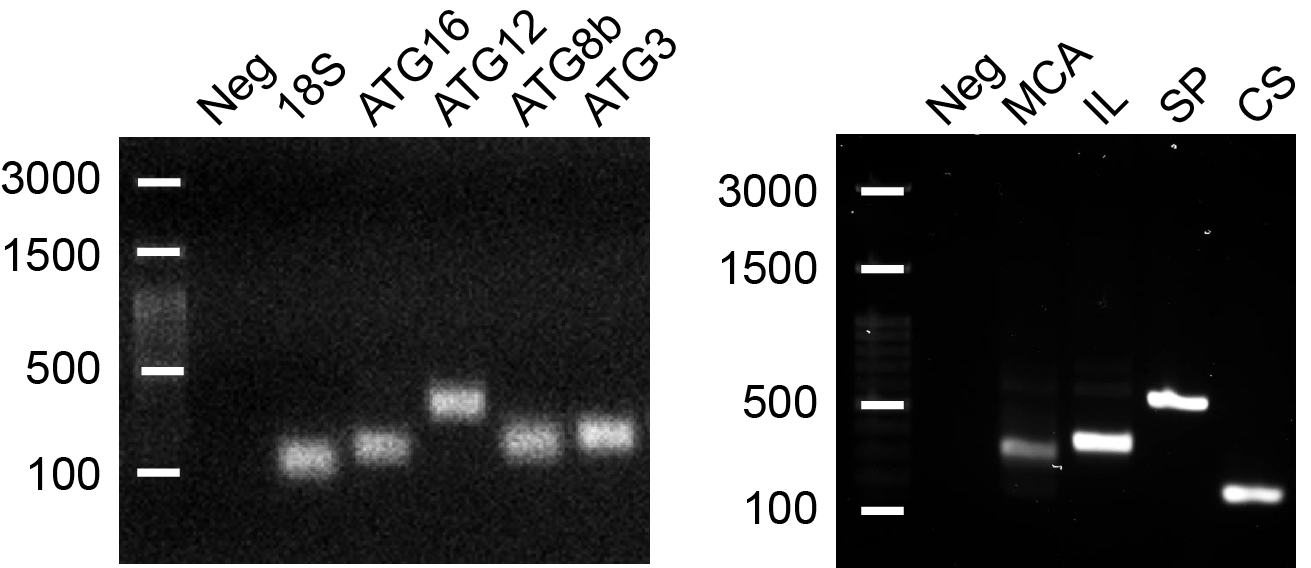


**Figure S4.** **Gel electrophoresis of PCR products.** Conventional PCR was performed to validate a primer specificity against *A. triangularis* DNA. The PCR product was run on 1.5% agarose gel. The primers targeting autophagy genes i.e. ATG16, ATG12, ATG8b, ATG3; encystation-related genes i.e. cellulose synthase (CS), serine proteinase (SP); apoptosis-related genes i.e. metacaspase (MCA), interleukin-1 converting enzyme-like protease (IL) including 18S rRNA were labelled on top of the gel. The first lane was a DNA ladder in bp (DM2300, SMOBIO^®^, Hsinchu, Taiwan) and the second lane was a negative control (Neg).


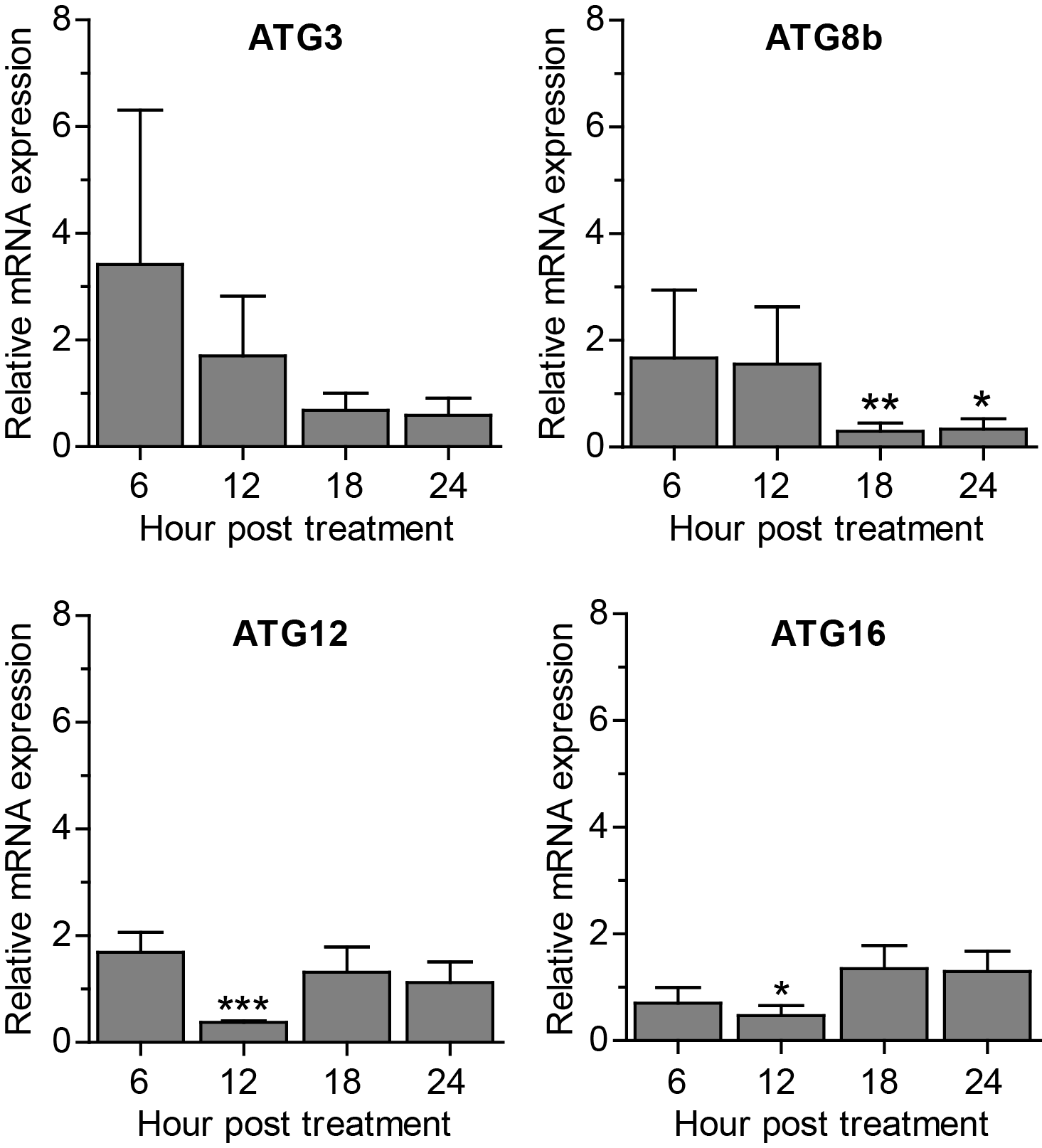


**Figure S5.** **Transcriptional expression of *A. triangularis* autophagy-related genes after 3MA treatment under starved condition.** The trophozoites were cultured in PAS supplemented with 5% glucose in the presence or absence of 1mM 3MA and incubated for 24 h. The parasites were harvested, analyzed mRNA level of ATG3, ATG8b, ATG12, ATG16 by qPCR, and expressed as a relative mRNA expression. 18S rRNA was included as a reference gene. The expression at time 0 h was set to 1. Data were obtained from 3 independent experiments. Bar graphs represent mean±SEM. *, *P*<0.05; **, *P*<0.01; ***, *P*<0.001.


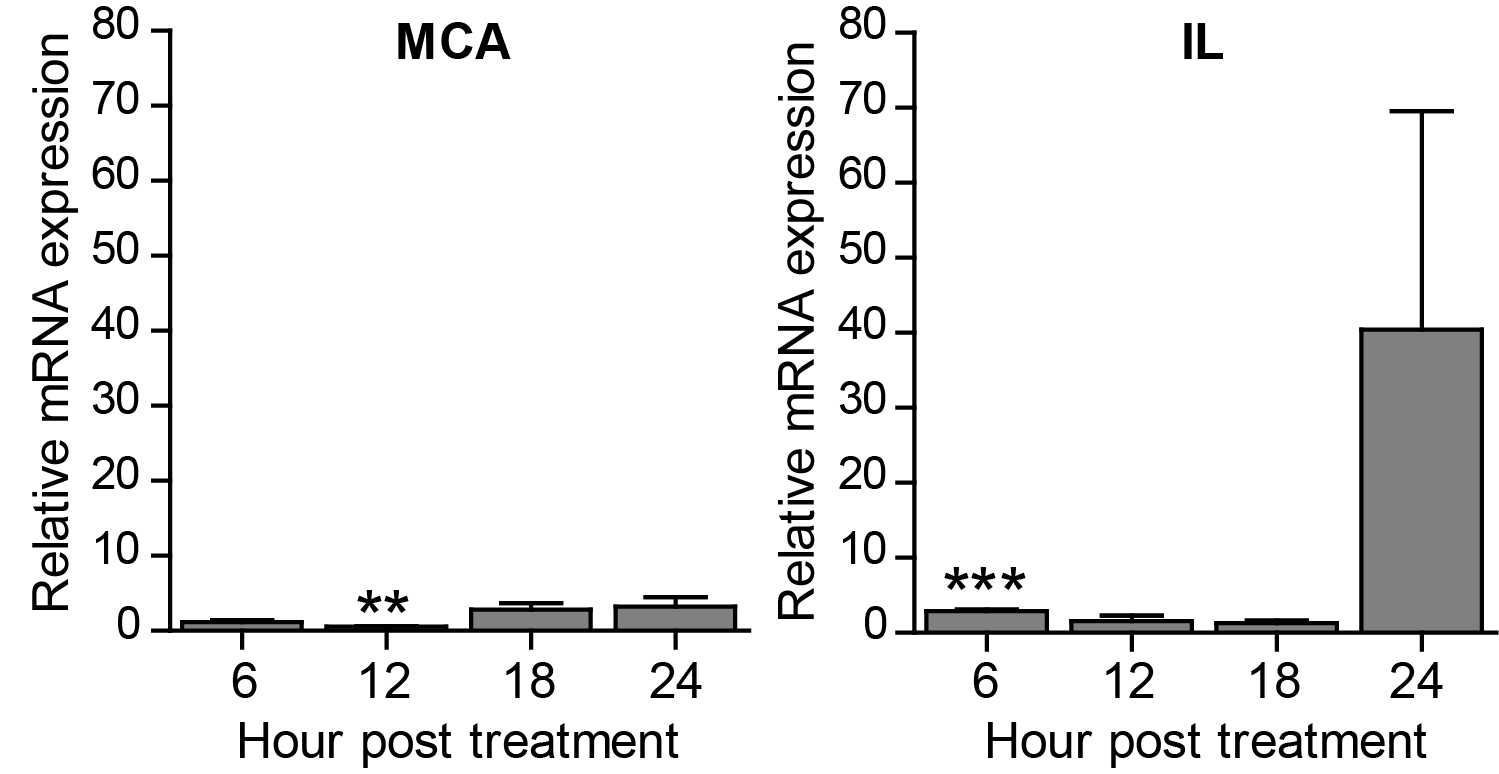


**Figure S6.** **Transcriptional expression of *A. triangularis* apoptosis-related genes.** Expression level of metacaspase (MCA) and interleukin-1 converting enzyme-like protease (IL) mRNAs were analyzed. The cDNA samples were shared with autophagy analysis. The qPCR was performed and 18S rRNA was included as a reference gene. Data were obtained from 3 independent experiments. Bar graphs represent mean±SEM. **, *P*<0.01; ***, *P*<0.001.

**Table S1**: Effect of curcumin in combination with chlorhexidine against *A.* *triangularis* trophozoites viability.

| **Curcumin**  **(µg/mL)** | | | **Percentage viability** | | |  |  |
| --- | --- | --- | --- | --- | --- | --- | --- |
|  | **250** | 7.8±0 | 7.8±0 | 7.8±0 | 7.8±0 | 7.8±0 | 7.8±0 |
|  | **125** | 44.7±3.7 | 50.0±3.7 | 50.0±3.7 | 50.0±3.7 | 42.1±3.7 | 7.8±0 |
|  | **62.50** | 57.8±3.7 | 52.6±3.7 | 50.0±3.7 | 47.3±7.1 | 44.7±3.7 | 7.8±0 |
|  | **31.25** | 86.8±1.4 | 57.8±9.8 | 71.0±0 | 55.2±0 | 47.3±7.1 | 7.8±0 |
|  | **15.62** | 97.3±9.8 | 84.2±3.7 | 86.8±1.4 | 55.2±0 | 50.0±3.7 | 7.8±0 |
|  | **0** | 100±0 | 92.1±3.7 | 81.5±7.4 | 65.7±3.7 | 52.6±3.7 | 5.2±3.7 |
|  |  | **0** | **1** | **2** | **4** | **8** | **16** |
| **Chlorhexidine (µg/mL)** | | | | | | | |

**Table S2**: Effect of curcumin in combination with 3MA against *A.* *triangularis* trophozoites viability.

| **Curcumin**  **(µg/mL)** | | | **Percentage viability** | | |  |  |
| --- | --- | --- | --- | --- | --- | --- | --- |
|  | **250** | 1.3±2.2 | 3.9±0 | 6.5±2.2 | 6.5±2.2 | 3.9±3.9 | 5.2±2.2 |
|  | **125** | 50.0±6.0 | 51.3±7.8 | 48.6±2.2 | 50.0±2.2 | 46.0±6.0 | 43.4±3.9 |
|  | **62.50** | 55.2±7.8 | 50.0±4.5 | 46.0±2.2 | 52.6±6.0 | 50.0±2.2 | 51.3±3.9 |
|  | **31.25** | 86.8±1.7 | 60.5±2.2 | 61.8±6.0 | 56.5±6.0 | 55.2±3.9 | 53.9±2.2 |
|  | **15.62** | 90.7±0 | 86.8±3.9 | 90.7±3.9 | 64.4±6.0 | 56.5±4.5 | 53.9±4.5 |
|  | **0** | 100±0 | 98.6±0 | 94.7±3.9 | 94.7±1.7 | 92.1±4.5 | 92.1±2.2 |
|  |  | **0** | **1.25** | **2.5** | **5** | **10** | **20** |
| **3MA (mM)** | | | | | | | |

**Table S3**: Effect of curcumin in combination with wortmannin against *A.* *triangularis* trophozoites viability.

| **Curcumin**  **(µg/mL)** | | | **Percentage viability** | | |  |  |
| --- | --- | --- | --- | --- | --- | --- | --- |
|  | **250** | 8.5±0 | 5.7±2.4 | 5.7±2.4 | 4.2±4.2 | 4.2±4.2 | 8.5±0 |
|  | **125** | 47.1±4.2 | 51.4±4.2 | 51.4±4.2 | 57.1±2.4 | 54.2±2.4 | 52.8±2.4 |
|  | **62.50** | 64.2±0 | 60.0±0 | 52.8±2.4 | 55.7±0 | 57.1±2.4 | 54.2±2.4 |
|  | **31.25** | 90.0±4.2 | 65.7±10.7 | 70.0±6.5 | 62.8±2.4 | 60.0±7.4 | 55.7±4.2 |
|  | **15.62** | 94.2±0 | 92.8±2.4 | 94.2±0 | 60.0±0 | 54.2±4.9 | 62.8±16.2 |
|  | **0** | 100.0 | 100.0±6.5 | 92.8±6.5 | 101.4±2.4 | 102.8±4.2 | 92.8±10.7 |
|  |  | **0** | **1.25** | **2.5** | **5** | **10** | **20** |
| **Wortmanin (µM)** | | | | | | | |

**Table S4**: List of primers for quantitative PCR.

| **Gene** | **Genbank Accession No.** | **Forward (F)** | **Reverse (R)** | ***Acanthamoeba* spp.** | **References** |
| --- | --- | --- | --- | --- | --- |
| **ATG3** | GU270859 | 5’-GCGCACGTACGATATCTCCATC-3’ | 5’-ATGAACACTTGGTTCGGCGTC-3’ | *A. castellanii* | *(Moon et al. 2011)* |
| **ATG8b** | KC524507.1 | 5’-CCGAGTTCCTGTGATCGTTGA-3’ | 5’-AGCTGTGTGACGGCAATATCG-3’ | *A. castellanii* | *(Moon et al. 2013)* |
| **ATG12** | HQ830265.1 | 5’-CCAGTCGAAGAGTACATGAAAGA-3’ | 5’-GCGAAGGAAGTCCACGA-3’ | *A. castellanii* | *(Kim et al. 2015)* |
| **ATG16** | FJ906697 | 5’-AGCTTGACTTCCATCACGCTGA-3’ | 5’-TGTTTGAGGTTGGCCCGAA-3’ | *A. castellanii* | *(Song et al. 2012)* |
| **Cellulose synthase (CS)** | EDCBI66TR | 5’-TCATCTACATGTTCTGCGCCC-3’ | 5’-CGATCCAGTTGTTGAGCATGC-3’ | *A. castellanii* | *(Aqeel et al. 2013; Moon et al. 2014)* |
| **Serine proteinase (SP)** | EU365404 | 5’-TCAAGGTGCTCGGATGCAAT-3’ | 5’-ATGTTAGCCACAGACTGCGTC-3’ | *A. healyi* | *(Moon et al. 2008)* |
| **Metacaspase (MCA)** | AF480890 | 5’-TCAGGTTAGGGACACGGATGG-3’ | 5’-CTCGTTGTAGACGTAGGGCAG-3’ | *A. castellanii* | *(Wu et al. 2018)* |
| **Interleukin-1 converting enzyme-like protease (IL)** | XM_004338552 | 5’-CCCGCAAGAAGAACAAGTGGAG-3’ | 5’-CGTCACTGCAGCCCGAAAACAT-3’ | *A. castellanii* | *(Wu et al. 2018)* |
| **18S rRNA** | - | 5′-TCCAATTTTCTGCCACCGAA-3′ | 5′-ATCATTACCCTAGTCCTCGCGC-3′ | *A. castellanii* | *(Song et al. 2012)*, *(Moon et al. 2008)* |

**Table S5**: *A. triangularis* DNA sequence by Sanger sequencing.

| **Target gene**  (Accession No.) | **Primer** | **DNA sequence** | **Product length (bp).** | **% Identity**^a^ |
| --- | --- | --- | --- | --- |
| **ATG12**  (HQ830265.1) | F | 5’-TGTTCCTCTCCAAGGTCATCGTGTGCACCGCGCCTCAACCTCGTCTGTGCTCGTGTCACT  GTGTAATTATGTTCAAGCCGTTGGTGGCGCCTCCGCGTTGAAGGTGCGAATGCCTCCCCC  CACCACTTCATCGAGCCTCATTCTGTCGTGCATTCGCTTCGTCTGCAAGCGAACTGCTGC  TCTTTTGTGATCTGTATGAGACGCTTATCGCTGGTGTCGCTTGTGCTCAGGTGAACAAGT  TCAAGCTGCAAGCCAAGGCCTCGTTCCAGTTCGTCGTGGACTTCCTTCGCAAT-3’ | 293 | 98.08 |
|  | R | 5’-GTTCCAGTCGAAGAGTACATGAAAGATGGAAAAGGTATGTTCCCTCTCCCACAGTCATGG  TGTGCACCGCCCCTCAACCTCGTCCTGTGTCTGTGCAGTTGTAATTATGATCAAAGCCGT  AGGTGGCGNCCGCCGCCGTCTGAAGTGTGACAGAACGCCGCTACCTCCCACCACTTCATC  AGAGCAGCCATNACTGTCTTGCATTGTCCTCCGTCTGCAACGCGACACATGCTGCTAGCT  ATTTGTGATGCTGTAGGAGACGCTTATCGCTGGTGTCGCTTGTTCTCAGGTGAACAAGTT  CAAGCTGCAAGCCAGCTGCGA-3’ | 321 | 100.00 |
| **Metacaspase (MCA)** (AF480890) | F | 5’-ACTTCCGGCTTGACTGCAGTCTAGTACTATCAGACGTGTCTCGGTCCACAGAAAGATTGC  GGAGATCCAATAAGCTTTTTTCTGGCTTCCTCTATCCGCCATTGACGAACATGGAGATCG  AGGGCCCCGCTCTGCTAAGAGGAAGAGGGTTGAGGAGGATGTGGAGGAGGGACCCACTGC  ATCTGCTCCAACTGAAACGCAAAACTCTCAAAATGGCGATGCCATTCTAATGAACCACTG  CTACTCGATGTGCTGCCCTACGTCTACAACGAGAGAGCTGCCCTACGTCTACAACGAGAC  TGCACTCGGATGTCTCCCCGACCTTTTGTCCGAATGCAGCTTCCGCCAGGGCTAGGGCCG  TTTGTTCTTTCTCAGCTGGGTGCATTTCTACGGTTTTTTTTGGTTCCACTTGTTGACTTG  ACATATTTGCTCGTTGCCTTTGCTGACCACCCCAACCGGGAACCTAAACTGCTAGCTGGC  GACTCATTTTGCGTTACCGCTGGCGAAGGCGTGCTCACGCTGCCCTACGTCTACAACTAGAAA-3’ | 543 | -^b^ |
|  | R | 5’- TTCAGGTTAGGGACACGGATGGTAAGTGTGTCATCCAACAGACTCCCATCCGCCTTTCGA  TTTATGCTGACGTGTGTGGCGTGTGGTGGCGTGTGGCGGCGTGTGGCGGCGTGTGGCGGC  GTGTGGCGTGTGGCGTGTGAGGGAATAGCTACGTGTGCTTGTTCGAAGACAGGCCGAGCC  TTTGCGAGGGATTCATTTGGGACGGAATCATCACGTCGGGCATCAAGTACACCGGTTGGA  AAGATCAGGTTAGGGACACGGATGGATTTCAGGTTAGGGACACGGATGGACGTCAGCGCA  GGATCGCGGGCAGGACGGGCAAATAAAAACACTTTTTTAGTCGACAGAAATAGTTCGGAA  AATGAATAATAATTTTTTTTGGCGTAATCAATCCGCAATTGACGAAGATGGAGATCGAGG  GCCAGCGTTCTGCGAAGAGGAAGAGGGTTGAGGTCGATGTGGAGGAGGGACACGCTGCAG  CGGCTCCAGCGGAGACACACTACGCACCAAATGGCGAAGCCGCGTCGAAGTAGCCAGTTATTTAG-3’ | 545 | -^b^ |

**Table S5 (Cont.)**: *A. triangularis* DNA sequence by Sanger sequencing.

| **Target gene**  (Accession No.) | **Primer** | **DNA sequence** | **Product length (bp).** | **% Identity**^a^ |
| --- | --- | --- | --- | --- |
| **Interleukin-1 converting enzyme-like protease (IL)**  (XM_004338552) | F | 5’-GAACTTCGCTTAGGATTCGTTGCTCTGCCTGATCTAACGACCGATCTTCCTTTCACACAG  CCAAGCTGGGCGATATGCTCATGGGCGTTTCCAGTTCGATGATGGGTTATAGGGGCACGG  ACGACTTTGGCTGCAAGACCACCGACCGCGAATACGACTACCAGGCCCATCTGAGTAGGA  TCTCTCAGGTACCCTACCCTTTCCACTGCTCCTTTCGGGCAAGTTTGACTCACCAGCTCG  ATGCGCATTCAAGCCGACGTGGTGATGTTTTCGGGCTGCAGTGACGAAA-3’ | 289 | 87.40 |
|  | R | 5’-CTAAAATAAACCGTAGATATGAGCCAGACAACTCACGCCTCGACAAAGGGTTTTTTTTTT  TTAATAGAAAGCAACAAACCACCACACTTGTGTGGGGAGAAGATACCTCCGCGCGTGTGC  GTCTCTAGCGTCTGGTCATACGGAAGGACAAACCCCTCAACGTAATTATGCATAAGTGTA  ATTAACAGCCCAGATGCCCGCAAGATGAACCACCGGTTATACGAGTGGGGTGCCACCAAC  CCGCGGCATTCCAGATACAATAGGGCATATTAACGTGGTGGGGCCCGCAAGAAGAACAAT  CCCGCAAGAAGAACAAGTGGAGCAGTACGCACAGCCATGCAGCTCGCCACGATTCGTGCA  TCTCCTGATCTAACGACCGATGTTCCTTTCACACAGCCAAGGTGGGCCACATGCTCATGG  GCGTTTCCAGTTGGATGATGGGTTATAGGGGCACGGACGACGGGGCGGGGAGACCACCGA  CCGCGAATACAAATACCAGGCCCATCTGAGTAGGATCTCTCAGGTAGCCTACCCTTTCCA  CGGCTCCTTTCAGGGCAAGTTGACTCTACCAGCTCGAGTGCATGCAGTCCGT-3’ | 592 | 86.61 |
| **18S rRNA** | F | 5’-AATGGAATGGAATAGGACCTGTCCTCCTATTTTCAGTTGGTTTTGGCAGCGCGAGGACTA  GGGTAATGATA-3’ | 71 | 98.48 |
|  | R | 5’-TGAAATTAGGAAGGAACGGGTCCTATTCCATTATCCCATGCTAATGTATTCCGGTGGCAG  AAAATTGGAAAATAGGAC-3’ | 78 | 92.75 |

^a^ The DNA sequences were blasted against *A. castellanii* in NCBI database.

^b^ No significant similarity found by NCBI-DNA blast (*A. castellanii* ATCC30011).

^#^ The DNA sequences of ATG3, ATG8b, ATG16, cellulose synthase, and serine proteinase were published by Rachasak Boonhok, *et al*. 2021 *(Boonhok et al. 2021)*.
